# Deep Learning of Cell Spatial Organizations Identifies Clinically Relevant Insights in Tissue Images

**DOI:** 10.21203/rs.3.rs-2928838/v1

**Published:** 2023-07-04

**Authors:** Shidan Wang, Ruichen Rong, Donghan M. Yang, Xinyi Zhang, Xiaowei Zhan, Justin Bishop, Clare J. Wilhelm, Siyuan Zhang, Curtis R. Pickering, Mark G. Kris, John Minna, Yang Xie, Guanghua Xiao

**Affiliations:** 1 Quantitative Biomedical Research Center, Peter O’Donnell Jr. School of Public Health, University of Texas Southwestern Medical Center, Dallas, Texas, USA; 2 Department of Pathology, University of Texas Southwestern Medical Center, Dallas, Texas, USA; 3 Department of Thoracic Oncology, Memorial Sloan Kettering Cancer Center, New York, New York, USA; 4 Department of Surgery, Yale School of Medicine, New Haven, CT, USA; 5 Hamon Center for Therapeutic Oncology Research, UT Southwestern Medical Center, Texas, USA; 6 Simmons Comprehensive Cancer Center, UT Southwestern Medical Center, Dallas, Texas, USA; 7 Department of Pharmacology and Internal Medicine, University of Texas Southwestern Medical Center, Dallas, Texas, USA; 8 Department of Bioinformatics, UT Southwestern Medical Center, Dallas, Texas, USA

## Abstract

Recent advancements in tissue imaging techniques have facilitated the visualization and identification of various cell types within physiological and pathological contexts. Despite the emergence of cell-cell interaction studies, there is a lack of methods for evaluating individual spatial interactions. In this study, we introduce Ceograph, a novel cell spatial organization-based graph convolutional network designed to analyze cell spatial organization (i.e. the cell spatial distribution, morphology, proximity, and interactions) derived from pathology images. Ceograph identifies key cell spatial organization features by accurately predicting their influence on patient clinical outcomes. In patients with oral potentially malignant disorders, our model highlights reduced structural concordance and increased closeness in epithelial substrata as driving features for an elevated risk of malignant transformation. In lung cancer patients, Ceograph detects elongated tumor nuclei and diminished stroma-stroma closeness as biomarkers for insensitivity to EGFR tyrosine kinase inhibitors. With its potential to predict various clinical outcomes, Ceograph offers a deeper understanding of biological processes and supports the development of personalized therapeutic strategies.

## INTRODUCTION

Cells are the fundamental building blocks of tissue architecture. They are organized into cellular components, which, in conjunction with the extracellular matrix, form tissue structures(1). Cell spatial organization refers to the arrangement, distribution, and interactions of cells within a tissue. This includes the relative positioning of different cell types, their morphological features, and the relationships among them. Cell spatial organization and tissue architecture offers critical insights into disease states. For instance, the crosstalk between cancer cells and stromal cells is essential for invasive growth and metastasis (2, 3). Fibroblastic and necrotic cells also play significant roles in tumor proliferation and invasion(4–6). The spatial heterogeneity of tumor-infiltrating lymphocytes (TILs) is associated with the molecular profile of tumors and patient prognosis(7, 8). Examining the spatial organization of multiple cell types within tumor tissues and their surrounding microenvironments is crucial for understanding how these cells assemble and interact to produce diverse functional outcomes. Recent studies in cancer researchhave highlighted the importance of investigating how cell spatial organizations impact cancer biology and tumor progression(8–10). Although recent approaches to modeling cell-cell spatial interactions and their effects on biomarker expressions have emerged(11), they have only considered the spatial distance between two cells. In contrast, the overall cell spatial architecture, such as cell types, cell structure alignments, and interactions among multiple cells, has been overlooked. Additionally, these methods have combined the influence of all neighboring cells without distinguishing individual cell-cell interactions. Consequently, there is a pressing need for computational methods capable of characterizing the complex cell spatial organization (i.e. cell location, proximity, and relationships between different cell types, such as tumor cells, stroma cells, and immune cells) in order to identify single-cell level features and spatial interactions with biological significance.

Advancements in imaging technology have made whole-slide image (WSI) scanning of tissue slides a common clinical practice, producing large volumes of high-resolution digital images that contain in-depth information about tissue structures and cell interactions. Furthermore, the recent development of deep learning algorithms in digital pathology has enabled the automatic identification and classification of millions of cells from WSIs(12, 13). Studying cell spatial organizations from these routinely available WSIs may enhance our understanding of how cells are spatially organized to perform biological functions and how this organization influences tumor histology, progression, and treatment response. Convolutional Neural Networks (CNNs) have been developed for tumor detection, subtype classification, and mutation status prediction(14) based on WSIs. However, existing image-based CNNs do not specifically focus on cell spatial organization features; instead, they work with raw images that include a mix of tissue structures and external factors, such as inconsistent staining and scanning conditions. Consequently, interpreting image features and identifying cell-cell interactions that predict patient clinical outcomes become difficult. Traditional deep learning models present further hurdles for interpretation by biologists or clinicians. The mathematical outputs of these models often lack inherent meaning, creating a need to translate these numerical vectors into diagnostic or prognostic models that humans can understand. This disconnection between accurate clinical outcome prediction and the identification of influential factors underscores the need for innovative deep learning structures. By explicitly modeling cell spatial organization, we can significantly enhance the interpretability of deep learning models. This advancement would not only deepen our biological understanding but also improve the efficiency and stability of our analyses.

In this study, we developed Ceograph, a cell spatial organization-based graph convolutional network specifically designed to handle cell spatial interaction information in tissue images. Ceograph utilizes a graph model to represent cell spatial organization and incorporates features related to nuclei morphology and distribution, such as unique cell-cell interactions for different types, relative locations, and structural similarities. By adopting this approach, the Ceograph method emulates the cognitive process of the human brain, which first identifies cell types, then takes into account cell-cell spatial interactions, and finally interprets cell spatial organization, all while excluding potential noise. By assessing the influence of individual cell morphology and interactions on diagnosis and clinical outcomes, Ceograph identifies image features with both driving effects and clear biological meanings, potentially yielding new biological and clinical insights.

We validated the performance of Ceograph in three applications: 1) lung cancer subtype classification, 2) malignant transformation prediction in patients with oral potentially malignant disorders (OPMD), and 3) treatment response prediction in lung cancer. Our results demonstrate that Ceograph achieves state-of-the-art performance in lung cancer subtype classification, surpassing both traditional machine learning models(15) and CNNs(16–20). Moreover, Ceograph can predict clinical outcomes across various tissue types. We also demonstrated the interpretability of Ceograph models through the following examples: 1) In lung cancer subtype classification, Ceograph identifies features that align with existing histopathology knowledge, showing that lung squamous cell carcinoma (SCC) has a more structured architecture than lung adenocarcinoma (ADC). 2) In OPMD patients, Ceograph reveals that a disruption in the structure of epithelial strata, apart from the stratum basale, is associated with a high risk of malignant transformation. 3) In lung cancer treatment response prediction, Ceograph finds that elongated tumor nuclei and decreased stroma-stroma closeness are linked to reduced survival benefits from lung cancer targeted therapy (Ttx), providing novel insights into the biological mechanisms of treatment insensitivity by demonstrating their contributions. This work represents the first study to develop interpretable deep cell spatial interaction learning models for clinical outcome prediction and identification of cell spatial organization features that predict clinical outcomes using tissue images.

## RESULTS

### A Cell Spatial Organization-based Tissue Image Analysis Pipeline

**Figure 1:**
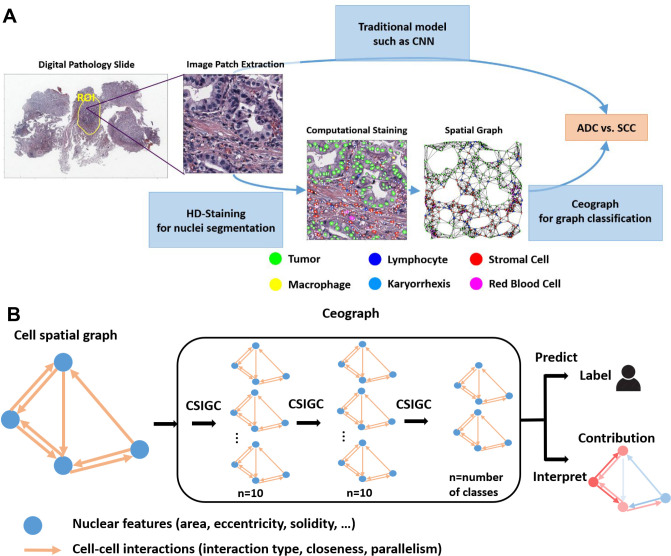
Illustration of using Ceograph for pathology image classification. **(A)** Flowchart of traditional image classification (upper arrow) and Ceograph (bottom arrow). The Ceograph method includes nuclei identification through HD-Staining, graph construction, and classification. The application to lung cancer pathological subtype classification is used as an example. The image patches are 1024×1024 pixels under 40X magnification. **(B)** Ceograph structure designed in this study, enabling both classification and model interpretation. ADC, adenocarcinoma; SCC, squamous cell carcinoma; CSIGC, cell spatial interaction-conditioned graph convolution.

Ceograph leverages cell spatial organization to model the impact of individual nuclei and cell-cell spatial interactions on biological phenotypes and clinical outcomes. To explicitly characterize cell spatial organization, we employed our Histology-based Digital (HD)-Staining model (12) to locate, segment, and classify each cell nucleus within a tissue image patch. Additionally, based on the segmentation results, we extracted well-defined nucleus morphology features, including size, shape, and orientation. Through this process, we transformed a tissue image into a spatial map of cells, where each cell’s nucleus location, cell type, and morphology are directly accessible for the deep learning architecture. Furthermore, individual cell-cell spatial interaction features are assessed as interaction types (determined by the type of a pair of nuclei), structural concordance (quantified as parallelism between a pair of nuclei), and interaction strength (quantified as nuclei spatial closeness). All this information serves as input for Ceograph (Figure 1A). In contrast, traditional CNN models use the entire image patch as input, which includes external noise and lacks explicit cell spatial organization features.

For a specific tissue image, the Ceograph method was applied through the following steps: (1) A set of image patches were randomly sampled from Regions of Interest (ROIs), which can be either manually annotated by pathologists or automatically determined by another computational algorithm. (2) Each nucleus within each image patch was segmented and characterized. In this study, the HD-Staining algorithm(12) was used for nuclei detection and classification. However, alternative nuclei or cell segmentation algorithms could also work, as long as the cell spatial locations were determined, making Ceograph a broadly applicable model for various spatial characterization technologies. (3) A cell spatial graph was constructed to represent the cell spatial organization in each image. The computed cell locations were used to determine the graph vertices and edges, with well-defined nuclei morphological features serving as vertex features (Supplemental Figure 1A), while cell types, concordances, and distances between pairs of nuclei used as edge features (Supplemental Figure 2). (4) Finally, a Ceograph model was trained by incorporating cell spatial organization information through three cell spatial interaction-conditioned graph convolutional (CSIGC) layers and another tumor cell-specific pooling layer (Figure 1B). The model was then used to predict the category, such as differential treatment response, for each graph (constructed from an image patch). By following these steps, the Ceograph method can effectively analyze tissue images, taking into account the cell spatial organization and interactions to provide meaningful insights into various clinical applications. By following these steps, the Ceograph method can effectively analyze tissue images, considering cell spatial organization and interactions to deliver valuable insights for a range of clinical applications.

In the Ceograph model, one CSIGC layer was designed to incorporate cell spatial organization by combining nuclei morphological features with their spatial interactions (Table 1 and Supplemental Figure 1). The CSIGC layer employs an attention mechanism that incorporates cell-cell spatial interaction information alongside nuclei morphological information, enabling Ceograph to focus on crucial cell-cell spatial interactions. Furthermore, the CSIGC layers intergrate information from neighboring nuclei while preserving cell-cell connection structures. This approach allows for a focus on nuclei of interest (such as tumor nuclei) in the pooling layer and contributes to Ceograph’s interpretability. By incorporating both morphological and spatial interaction features, the CSIGC layer enhances the model’s ability to analyze complex tissue images and derive meaningful insights from cell spatial organization.

### Ceograph Accurately Classifies Pathology Subtypes in Testing and Independent External Validation Datasets

We evaluated Ceograph’s performance against traditional image-based deep learning models in a classification task for lung adenocarcinoma (ADC) vs. squamous cell carcinoma (SCC) pathology images. For each lung cancer WSI, we randomly sampled 1,024×1,024-pixel image patches (with 0.25 microns per pixel) from pathologist-annotated tumor regions. Using TCGA lung cancer datasets (see Dataset Section), we created training, validation, and testing sets containing 52,189 image patches from 593 slides, 7,328 image patches from 83 slides, and 15,442 image patches from 172 slides, respectively (Supplemental Figure 3), with at least 20 tumor cells per patch. The independent National Lung Screening Trial (NLST) dataset, with 36,400 graphs from 496 slides, served as an external validation dataset.

**Figure 2:**
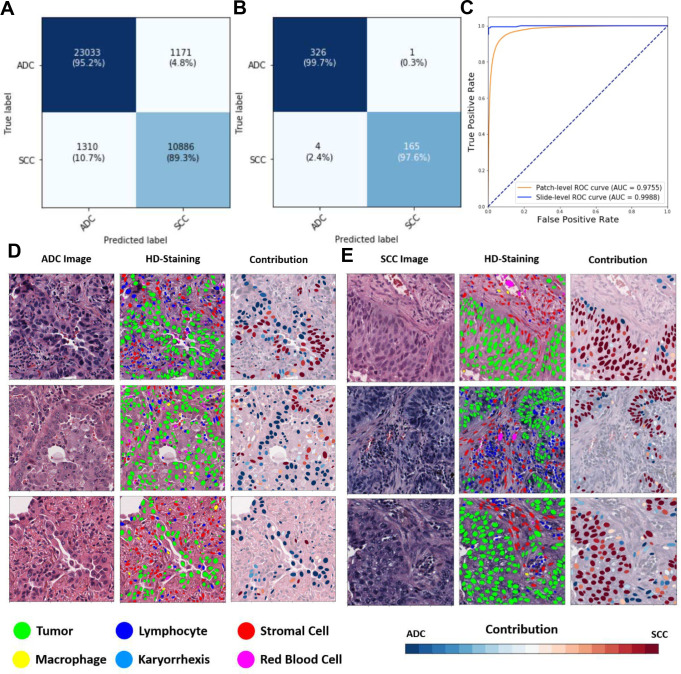
Performance of Ceograph in lung histology subtype classification. **(A-C)** Classification performance in the NLST independent testing dataset: image patch-level confusion matrix **(A),** slide-level confusion matrix **(B),** ROC curve **(C). (D-E)** Visualizing the contribution of each tumor nucleus to the final histological classification. Three examples from ADC **(D)** and SCC **(E)** are selected, respectively. Original image (1^st^ column), HD-Staining (2^nd^ column), and cellular-level contribution to subtype classification (3^rd^ column) are plotted. For better illustration of nuclei features, the mask of each cell is reconstructed from nuclei morphological features extracted by HD-Staining. Blue: contributing to ADC subtype; red: contributing to SCC subtype.

We first analyzed each image patch using HD-Staining to segment and classify six nuclei types: tumor nuclei, stroma nuclei, lymphocyte nuclei, red blood cells, macrophage nuclei, and karyorrhexis (see the Methods Section). We then derived cell spatial graphs from the HD-Staining analysis results (see the Methods Section) and trained a Ceograph model to predict lung cancer subtypes in the TCGA training set. We systematically assessed Ceograph’s performance in ADC vs. SCC classification tasks for both the TCGA testing dataset and the independent NLST dataset at image-patch and slide levels. In the TCGA testing dataset, patch-level accuracy reached 94.5% with an Area under Curve (AUC) of 0.986; slide-level accuracy achieved 100% with an AUC of 1.000 (Supplemental Figure 4). In the NLST external validation dataset, patch-level accuracy was 93.2% with an AUC of 0.976, while slide-level accuracy was 99.0% with an AUC of 0.999 (Figure 2A–C). In the lung cancer pathology subtype classification task, Ceograph outperformed all other CNN-based deep learning models and traditional feature-based machine learning methods (Table 2).

To further evaluate Ceograph classification performance, a CNN model with the popular ResNet101 architecture(21) and a logistic regression model were trained and evaluated on exactly the same image patches, respectively (Table 2). As expected, ResNet101 performed similarly to previous reports (0.966 slide-level AUC, Supplemental Figure 5). Moreover, it is noteworthy that the logistic regression model using the image patch-level averaging of image features also achieves a comparable performance to CNN-based deep learning models (0.914 slide-level AUC in the NLST dataset, Supplemental Figure 6), which validated the informative and interpretable nuclear features derived by HD-Staining. Importantly, the superior performance of Ceograph indicated that the cell organization information used in the cell spatial graph was critical to improving tumor subtype classification accuracy (Table 2). The direct comparison of different prediction methods once again demonstrated the superior performance of Ceograph, which benefits from explicit usage of cell spatial organization information based on histopathological knowledge.

### Understanding the Prediction Mechanism of Ceograph

Neural networks are frequently criticized as a “black box” due to their complex structures and massive number of parameters (up to 44.5 million(22)). Therefore, it is both challenging and intriguing to understand how a deep neural network works. The Ceograph method uses graph structure by modeling each cell nucleus as a vertex and a pair of neighboring cell nuclei as an edge (Supplemental Figure 2). Ceograph represents cell nuclei morphological features as vertex features, while incorporating cell types, postures, and distances between nuclei as edge features. The contributions of these vertices and edges are then summarized through the use of CSIGC layers within the neural network. As a result, Ceograph effectively harnesses the intricate and nuanced patterns in cell spatial organization to characterize tissue images and to improve prediction accuracy. By modeling image-derived features, we visualized cellular level contributions to Ceograph’s classification of ADC/SCC to understand how different cell spatial organization patterns affect the histological determination by Ceograph. The contribution of each cell to the ADC/SCC classification was calculated (detailed in the Methods) and depicted in Figure 2D–E. Notably, regions with sheets of polygonal cells contributed to SCC classification, reflecting the histopathological feature of the SCC subtype(23). As a result, the cell-level contribution heatmap effectively highlights typical cell organization patterns for ADC and SCC.

**Figure 3:**
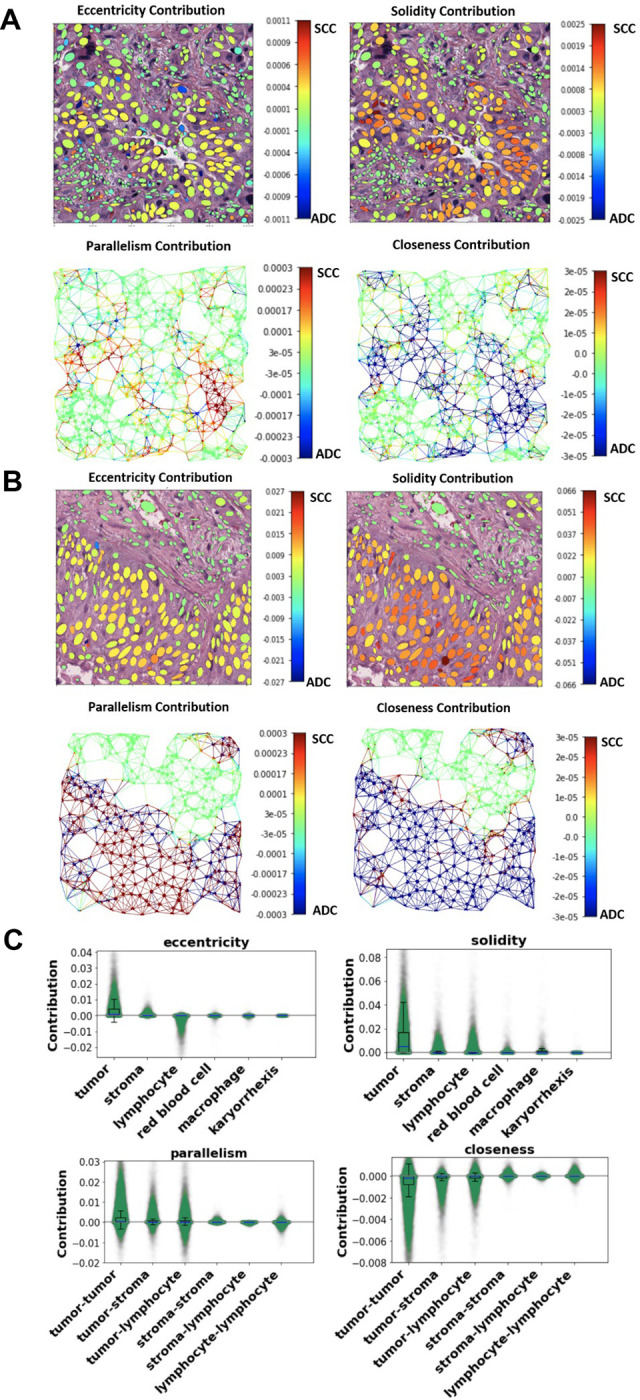
Visualization of the working mechanism for Ceograph. **(A-B)** Contributions of input features of individual cells and edges are calculated as partial derivatives of an objective function of being predicted as ADC with respect to the input features, which include both nuclei morphologies and edge attributes. The contributions are plotted for example ADC image patch (**A**) (original image in Figure 2D) and SCC image patch (**B)** (original image in Figure 2E), respectively. For better visualization, the contribution of edge attributes is colored in the graph edges of the upper panels. Redder color represents positive partial derivative and contribution to SCC subtype, while bluer color represents negative partial derivative and contribution to ADC subtype. **(C)** Boxplots to summarize feature contributions across the entire NLST dataset. Positive value indicates contribution to SCC subtype, while negative value indicates contribution to ADC subtype.

To better understand how Ceograph makes predictions, we analyzed the impact of different cell distribution features. We visualized the contribution of each feature to the final classification of ADC vs. SCC tumors in Figure 3A–B and found that higher values of eccentricity and solidity of tumor cells in both ADC and SCC graphs (Figure 3A and 3B) increased the likelihood of a region being classified as SCC. This aligns with our prior knowledge that SCC tumor cells have higher eccentricity and solidity than ADC tumor cells (Supplemental Figure 7). Additionally, we observed that the contribution from other cell types was considerably lower (both p-values < 0.001, Figure 3C). Furthermore, we discovered that cell-cell spatial interaction features, such as nuclear parallelism (Supplemental Figure 2) and nuclear closeness (inverse of distance between nuclei centroids as described in the Method Section), were critical in predicting the pathological subtype. Interestingly, we found that spatial interactions among tumor cells were more significant than interactions among other cell types (all p-values < 0.001, Figure 3C). These results align with pathological observations that SCC tumor cells have a more structured architecture with elongated nuclei shapes compared to ADC tumor cells, confirming the interpretability of Ceograph.

### Ceograph Predicts Risk of Malignant Transformation of OPMD

**Figure 4:**
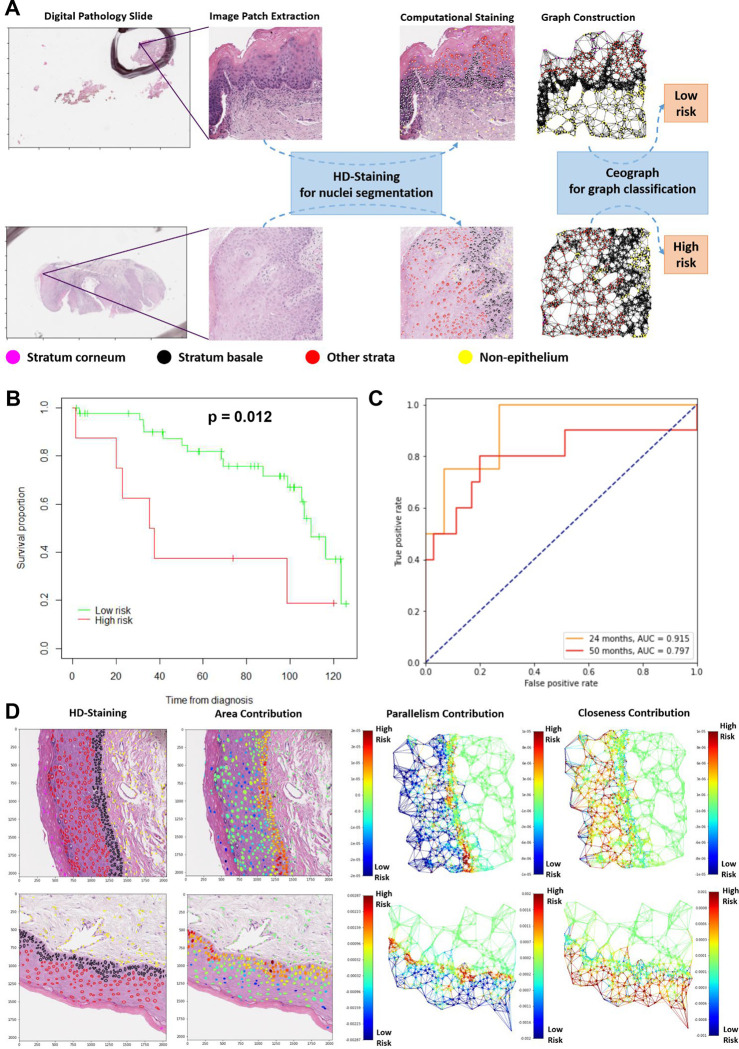
Prognostic value of Ceograph in predicting risk of malignant transformation. **(A)** Flowchart of risk prediction from pathology slides of Oral Potentially Malignant Disorders (OPMD) patients. The image patches are 2048×2048 pixels under 40X magnification. **(B)** Kaplan-Meier curves of predicted high- vs. low-risk patient groups in the OPMD2 testing set. **(C)** ROC-curves of predicting malignant transformation events at 24 months and 50 months, respectively, in the OPMD2 testing set. **(D)** Model interpretation of Ceograph via partial derivatives of an objective function of being predicted as low-risk class. Redder color represents contribution towards higher risk score with increasing the input value (e.g., area). Upper panel, an example image patch predicted as low-risk from a patient with good prognosis (CFS > 71.5 months); bottom panel, an example image patch predicted as high-risk from a patient with poor prognosis (CFS = 20.2 months).

To explore Ceograph’s generalizability across different tissues and clinical questions, we applied the method to a prognostic prediction task. Specifically, oral potentially malignant disorders (OPMD) are a group of oral cavity mucosal diseases with a risk of progressing to oral squamous cell carcinoma. Traditionally, risk assessment involves a combination of clinical and histologic evaluation. Leukoplakia, a common type of OPMD, is assigned a histologic grading score, which is supposed to correlate with its risk of progression. However, significant intra- and inter-observer variability in dysplasia grading leads to inconsistencies and uncertainties in risk assessment and treatment planning. In this study, we employed the Ceograph method to predict the risk of malignant transformation in skin tissue for OPMD patients (Figure 4A).

We utilized the morphological characteristics and spatial organization of the four major nuclei types within skin tissue: stratum corneum, stratum basale, other strata, and non-epithelium. Subsequently, we trained a prognostic Ceograph model using the OPMD1 dataset (refer to the Method Section for a detailed description of the OPMD1 and OPMD2 datasets) to distinguish between high-risk (defined as Cancer Free Survival [CFS] ≤ 42.3 months) and low-risk (defined as CFS > 68.5 months) groups (Supplemental Figure 8). In the OPMD2 independent testing set, the predicted high-risk group demonstrated significantly shorter CFS compared to the low-risk group (Figure 4B, p = 0.012; high-vs. low-risk, Hazard Ratio [HR] = 3.17, 95% Confidence Interval [CI] 1.22 – 3.22). Moreover, the prognostic Ceograph effectively predicted the likelihood of 24-month CFS (AUC = 0.915) and 50-month CFS (AUC = 0.797, Figure 4C). The modest reduction in AUC for the 50-month prediction, compared to the 24-month prediction, may be attributed to the accumulation of additional confounding factors over the longer prediction timeline. These results suggest that Ceograph can identify OPMD patients at a higher risk of cancer by discerning the differences in cell spatial organization between OPMD tissues with a high risk of progressing to cancer and those with a low risk.

To further translate the information learned by Ceograph into pathological knowledge, we visualized the contributions of cellular-level features to Ceograph’s risk assessment of malignant transformation (Figure 4D). Feature contributions were consistent across graphs for both low-risk (Figure 4D, upper panel) and high-risk (Figure 4D, bottom panel) patients, and are summarized in Supplemental Figure 9. Malignant transformation risk increases with larger nuclear areas in the stratum basale, aligning with previous pathological knowledge that increased nuclear area is a characteristic of dysplasia (24). Comparative analysis of the OPMD1 dataset also shows that the nuclear area of stratum basale (p = 2.2×10^−19^), rather than any other stratum, was significantly higher in the high-risk group than in the low-risk group (Supplemental Figure 10). To better comprehend the impact of cell spatial interactions and organization on malignant transformation risks, we visualized the contributions from edge features (Figure 4D) and summarized them (Supplemental Figure 9). In epithelial strata other than the stratum basale, reduced parallelism and increased closeness were associated with higher risk, consistent with pathological observations of disrupted cellular architecture in epithelial dysplasia. Our comparison studies confirmed decreased parallelism in epithelial strata within the high-risk group, and nuclear parallelism in other strata showed the most significant difference between high- and low-risk groups (p = 2.9×10^−34^, Supplemental Figure 10).

In summary, the distinct contributions of cells in different epithelial layers demonstrate that Ceograph accurately and interpretably captures the morphological and structural characteristics of various epithelial strata.

### Ceograph Predicts EGFR TKI Ttx Response

**Figure 5:**
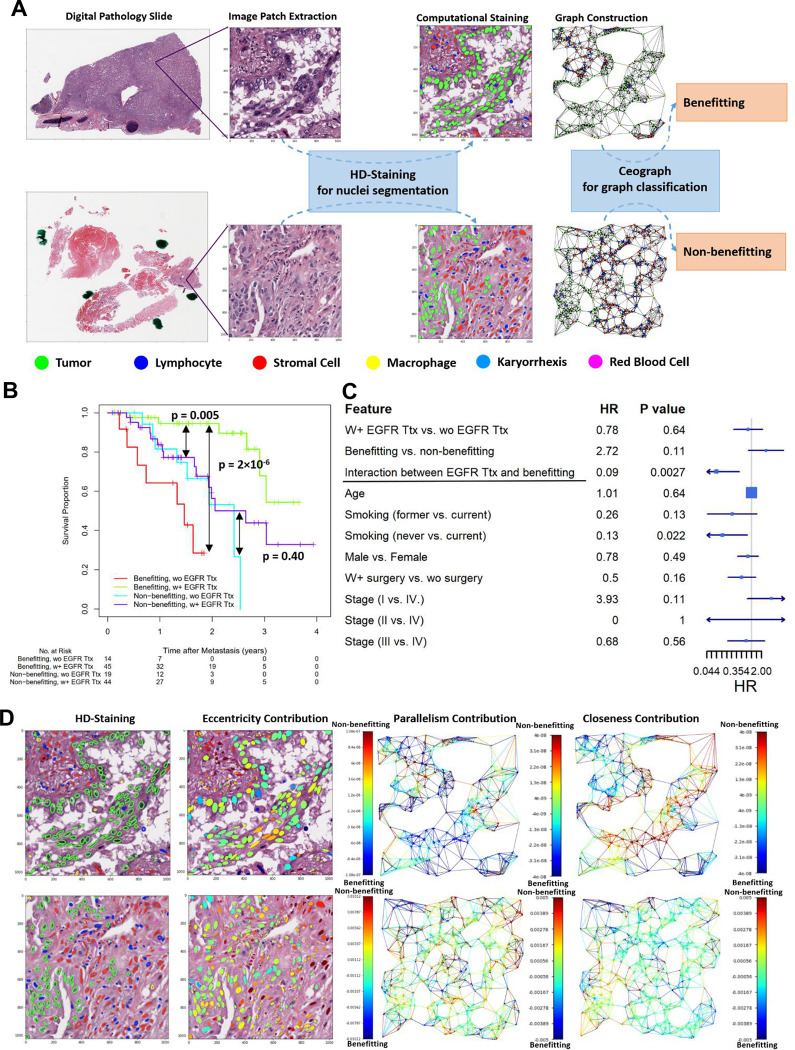
Predictive value of GCN in EGFR TKI Target therapy (Ttx) response prediction. **(A)** Flowchart of EGFR TKI treatment response prediction from pathology slides of lung adenocarcinoma patients. **(B)** Visualize patient survival outcomes using Kaplan-Meier curves in the Lung Cancer Mutation Consortium 2 (LCMC2) testing dataset. Patients with EGFR mutations are grouped based on their predicted benefitting score as well as whether or not they received EGFR TKI Ttx. In patients who received EGFR TKI, the significantly better survival outcome of the predicted benefitting group over the non-benefitting group (p value = 0.02) demonstrates the prognostic value of the benefitting score. The improved survival outcome for patients who received EGFR TKI Ttx is detected only in the predicted benefitting group (p value = 5 × 10^−6^) rather than the non-benefitting group (p value = 0.30), demonstrating the predictive value of the benefitting score. **(C)** Forest plot of multivariate Cox Proportional Hazard model adjusted by potential confounders, including age, smoking status, gender, surgery, and stage. **(D)** Model interpretation of the predictive Ceograph via partial derivatives of an objective function of being predicted as benefitting group. Redder color represents contribution of increased value (e.g., nuclear eccentricity) to decreased benefit. Upper panel, an example image patch predicted as benefitting; bottom panel, an example image patch predicted as non-benefitting.

Although epidermal growth factor receptor (EGFR) tyrosine kinase inhibitor (TKI) Targeted therapy (Ttx) is considered first-line therapy for patients with metastatic lung cancer with *EGFR* mutation, only 60% to 80% of patients with sensitizing *EGFR* mutations respond to such therapy(25, 26). Developing predictive models that can accurately forecast cancer patients’ response to treatment before its actual administration is of great clinical importance. This approach can not only enhance treatment efficacy but also minimize adverse side effects and save valuable time and resources by allowing clinicians to bypass ineffective treatments. Given Ceograph’s state-of-the-art performance in histological classification and its success in prognostication, we further evaluated the value of Ceograph in predicting response to EGFR TKI Ttx using the tissue pathology slides collected before the treatment, which is widely regarded as a more challenging task than prognostication. A pipeline similar to Figure 1A was designed and applied to the task (Figure 5A, Supplemental Figure 11). The predictive Ceograph was trained to predict the benefitting score for each input cell spatial graph in the Lung Cancer Mutation Consortium 1 (LCMC1) dataset where all patients with *EGFR* mutation were treated with EGFR TKI Ttx. Patients with overall survival (OS, defined as time from diagnosis of metastatic disease to death or last contact) > 31 months were labeled as benefitting; otherwise, patients with OS ≤ 31 months were labeled as non-benefitting.

We applied the predictive Ceograph to patients with *EGFR* mutation in the independent LCMC2 testing dataset. First, the prognostic value of the predicted benefitting score was confirmed in the patients who received EGFR TKI Ttx, with the predicted benefitting group showing significantly better OS than the non-benefitting group (Figure 5B, p = 0.005; benefitting vs. non-benefitting, HR = 0.30, 95% CI 0.12–0.79). More importantly, the predictive value of the Ceograph analysis was assessed by comparing the benefits of EGFR TKI Ttx in the benefitting group and non-benefitting groups. Within the predicted benefitting group, patients who did not receive EGFR TKI Ttx showed significantly worse OS than patients who received EGFR TKI Ttx (Figure 5B, p = 2×10^−6^; wo Ttx vs. w+ Ttx, HR = 16.60, 95% CI 3.43 – 80.33). In contrast, within the predicted non-benefitting group, no significant survival difference between Ttx-treated and non-treated patient groups was observed (Figure 5B, p = 0.40; wo Ttx vs. w+ Ttx, HR = 1.44, 95% CI 0.59 – 3.49). After adjusting for potential clinical confounders, including age, gender, smoking status, surgery, and stage at diagnosis, a high benefitting score calculated by Ceograph was still predictive of prolonged OS in patients with *EGFR* mutations who received EGFR TKI Ttx (p = 0.0027 for the interaction term between benefitting group and EGFR TKI Ttx, Figure 5C). Given the diversity of EGFR TKI Ttx response^33,34^, our predictive model is important for personalized treatment selection to maximize the benefit of EGFR TKI Ttx.

To understand the relationship between cell spatial organization features and sensitivity to EGFR TKI Ttx, we interpreted the predictive Ceograph by determining how increasing individual input features would affect the predicted benefitting score. We quantified the graph feature contributions, with a positive value indicating a contribution to the non-benefitting group (Figure 5D, Supplemental Figure 12). Interestingly, increasing tumor cell eccentricity generally raised the likelihood of being predicted as non-benefitting from EGFR TKI Ttx. This observation aligns with comparative study results in the LCMC1 dataset, which showed significantly higher tumor cell eccentricity in the non-benefitting group compared to the benefitting group (p = 1×10^−52^, Supplemental Figure 13). The observation that increasing tumor cell eccentricity generally leads to a higher likelihood of being predicted as non-benefitting from EGFR TKI Ttx suggests a connection between the elongated morphology of tumor nuclei and the epithelial-mesenchymal transition (EMT) process (27). The EMT process has been associated with EGFR TKI-resistant tumors (28), and thus, a higher tumor cell eccentricity in the non-benefitting group may indicate EMT activity, providing a possible explanation for the observed resistance. Furthermore, the model revealed that decreased stroma-stroma closeness generally correlated with an increased likelihood of non-benefitting from EGFR TKI Ttx, consistent with observations in the comparative study (p = 5×10^−48^, Supplemental Figure 13). Decreased stroma-stroma closeness may suggest the activation of cancer-associated fibroblasts (CAFs) with increased cell size, which has been linked to tumor progression and poor prognosis (29). This implies an additional role for CAF activation in EGFR TKI resistance.

## DISCUSSION

In this study, we developed Ceograph, a novel graph-based deep learning model to characterize cell spatial organization in tumor tissues. Ceograph captures the subtle but coordinated changes in cell spatial organization and predicts patient outcomes with state-of-the-art performance in histology classification, as well as in prognostic and predictive tasks. To the best of our knowledge, this is the first study to develop comprehensive and rigorous models to characterize cell spatial organization using graph models at the single-cell level.

Using the proposed Ceograph model, we demonstrated that cell spatial organization features, including cell spatial distribution, cell-cell interaction, and nuclei morphological features at different locations, contain sufficient information for various patient outcome prediction tasks. These tasks encompass predicting tumor histological subtypes, malignant transformation, and therapeutic response. Our findings highlight the importance of cell spatial organization in distinguishing tumor subtypes and patient outcomes.

In this study, we designed the Ceograph model to use nuclei morphological and distribution features as input based on pathological knowledge, mimicking the human brain’s hierarchical image recognition and classification process. The model first “recognizes” cells using the HD-Staining model and then discerns comprehensive but subtle patterns in cell organization and morphological characteristics to classify tissue into categories associated with histology subtype or potential patient outcomes. This “cell recognition” step reduces the effect of variations in staining or lighting conditions, improving stability in the “classification” step.

The proposed Ceograph model represents a successful attempt to combine human knowledge with a neural network. As a result, the Ceograph model outperforms traditional CNN models while utilizing far fewer parameters. The proposed histology classification Ceograph, in particular, has only 9,912 parameters, which is roughly 1/5000^th^ of the popular ResNet101 model (#parameters = 44.5 million)(22) and 1/2500^th^ of the InceptionV3 model (#parameters = 23.9 million)(30). Decreasing the number of parameters improves portability, computational load, and interpretability while reducing the risk of overfitting. Although the Universal Approximation Theory(31) proves that neural networks can approximate any Borel measurable functions given enough hidden layers, large numbers of trainable parameters have long been contradictory with the constraints of limited training samples. Increasing signal-to-noise ratio and utilizing existing knowledge are important for resolving this contradiction, in addition to collecting and augmenting training data.

Understanding how a neural network makes a decision is important. Guided by the occlusion- and gradient-based methods, which have been utilized to estimate marginal attributes of an input feature applied to CNNs(32, 33), we propose that calculating partial derivatives of output with regard to each individual input feature is a simple and effective way to understand the contribution of each feature to the decision making process of Ceograph. The consistency of knowledge generated by Ceograph and direct comparative study demonstrates the validity of this method. More importantly, the results showed that Ceograph models are interpretable and lead to biological insights into how cells assemble and interact to produce diverse functional consequences in multiple contexts.

One limitation in this study is that the proposed Ceograph uses only nuclei features rather than cytosolic or plasma membrane features. Although cellular architecture is an important factor in the subtyping process by pathologists, cytosolic features are also helpful. For example, the cytosol morphology of signet ring ADC and mucinous ADC differs(34), and the presence of obvious plasma membrane is a marker for SCC. Although we demonstrated that the morphological features and nuclei distributions are useful for distinguishing between ADC and SCC, predicting risk of malignant transformation of OPMD patients, and predicting treatment response to EGFR TKI Ttx, combining cytosolic and membranous features may be useful in other tasks.

## METHODS

### Dataset

#### TCGA and NLST datasets for histology classification

Pathology images that support the lung cancer subtype classification results of this study are available online in TCGA (https://wiki.cancerimagingarchive.net/display/Public/TCGA-LUAD) and NLST (https://biometry.nci.nih.gov/cdas/nlst/). The H&E-stained pathology images (40X), together with the corresponding clinical data, were obtained from the NLST and TCGA cohorts: 469 pathology slides for 422 lung ADC patients and 380 pathology slides for 380 lung SCC patients were acquired from the TCGA Lung SCC dataset; 327 pathology slides for 193 lung ADC patients and 169 pathology slides for 93 lung SCC patients were acquired from the NLST dataset (there could be multiple pathology slides for a single patient). To refine our analysis within the tumor region, the tumor Region of Interest (ROI) for each of the pathology slides were labeled and confirmed by pathologists using the ImageScope software (Figure 1A).

#### OPMD1 and OPMD2 datasets for malignant transformation prediction

Patients with baseline OPMD were biopsied and followed at the Department of Head and Neck Surgery at The University of Texas MD Anderson Cancer Center (MDACC). The OPMD pathology images, reviewed by expert pathologists and scanned at 40X, were received in two successive batches two years apart, named OPMD1 and OPMD2 datasets respectively. Patients who had concomitant cancer were excluded from the OPMD datasets. With this criterion, 23 pathology slides from 23 patients were collected from the OPMD1 dataset and 53 pathology slides from 53 patients were collected from the OPMD2 dataset. Cancer-free survival (CFS) time was used to quantify risk of malignant transformation and defined as time from diagnosis of OPMD to diagnosis of oral cancer, or last follow-up. The maximum follow-up time was 126 months.

#### LCMC1 and LCMC2 datasets for EGFR TKI Ttx response prediction

The LCMC1(35) and the LCMC2(36) datasets are multi-institutional datasets established to study different oncogenic drivers and corresponding target therapies. Patients who had stage IV or recurrent lung ADC, Southwest Oncology Group performance status 0, 1, or 2, more than 6 months of expected survival, and tissue adequate for molecular analyses were considered eligible for LCMC1 and LCMC2. Biopsy slides and clinical information for patients who carried *EGFR* mutation were collected for this study. 115 biopsy slides from 98 patients were collected from the LCMC1 dataset and 137 biopsy slides from 126 patients were collected from the LCMC2 dataset. Patient characteristics are summarized in Supplemental Table 1.

### Nuclei Segmentation and Classification Using HD-Staining

Identification and classification of different cell types is a prerequisite for constructing cell graphs. An automatic deep learning model, HD-Staining, was trained to identify 6 different cell types in the lung cancer microenvironment: tumor cells, stromal cells, lymphocytes, red blood cells, macrophages, and karyorrhexis(37). The model was directly applied to image patches within tumor ROIs of pathology images (Figure 1A) and produced information about each identified nucleus, including centroid position, cell type, confidence of prediction, nuclear orientation (defined as the angle between x-axis and major axis of nucleus), and 10 well defined nuclear morphological features (area, convex area, eccentricity, extent, filled area, major axis length, minor axis length, perimeter square divided by area, perimeter, and solidity). The model is available online as a web-server at http://lce.biohpc.swmed.edu/maskrcnn/. Since HD-Staining was trained on pathology slides at 40X magnitude, pathology images at 20X magnitude were resized to 40X using SR-GAN(38). For 40X images, the spatial resolution is 0.25 microns per pixel (mpp).

In order to characterize the epithelial microenvironment for OPMD patients, we further generalized HD-Staining to oral epithelial tissues. The epithelial HD-Staining was trained to identify four different cell types: stratum corneum, stratum basale, other stratums, and non-epithelium. The same set of information as described before was extracted for each identified nucleus.

### Graph Construction Using k-Nearest Neighbors

The spatial organization of all cells within an image patch naturally fits in the graph concept. Thus, a directed graph was constructed for each image patch using k-Nearest Neighbors based on Euclidean distance with the direction pointing from a cell to its neighbors (Figure 1A). K was set to 8 to cover the adjacent neighbors of each nucleus. Each graph consisted of two components: nodes (representing nuclei) and edges (representing spatial interactions among nuclei). To further describe cell types, morphological features, and cell-cell spatial interactions as a graph, two feature matrixes were defined for nodes and edges, respectively. The node feature matrix contained 11 features: confidence of prediction and the 10 aforementioned morphological features generated by HD-Staining. The node features were globally centered and scaled before being fed into the Ceograph model. The edge feature matrix contained 3 features: categorical edge type based on cell types of starting node and ending node (yielding 6 * 6 = 36 edge types for 6 cell types in lung cancer microenvironment and 4 * 4 = 16 edge types for 4 cell types in epithelial microenvironment), nuclear parallelism defined as the absolute value of cosine of angle between major axes of the starting node and the ending node (a greater value indicates greater parallelism, Supplemental Figure 2), and nuclear closeness defined as the reciprocal of Euclidean edge length in pixels.

### Lung ADC vs SCC classification using Ceograph

We hypothesized that instead of directly using images, which are extremely high-dimensional, a much simpler graph representation of cell spatial organization and nuclei orientation would be informative enough to distinguish the two main histological subtypes of lung cancer, ADC and SCC. Thus, a GCN, Ceograph, was designed to utilize graph data instead of a CNN, which is only able to analyze structured data (Figure 1B). Ceograph was constructed with three cell spatial interaction-conditioned graph convolutional (CSIGC)(39) followed by a Subgroup Mean Pooling layer and a Softmax layer. Through the Subgroup Mean Pooling layer, only features for tumor nuclei nodes were averaged to focus on contributions from tumor nuclei. The computation algorithm of a CSIGC layer is illustrated in Supplemental Figure 1 and Table 1.

The classification Ceograph model was trained, validated, and tested using the TCGA dataset, and independently tested in the NLST dataset. To construct the training/validation/testing datasets, 100 1,024×1,024 pixels image patches were extracted from the ROI of each pathology slide (Supplemental Figure 3) and transformed into 100 graphs individually; only graphs containing at least 20 tumor cells were considered as informative enough to be classified as ADC/SCC and kept in the datasets. The TCGA ADC dataset contains 40,971 graphs (28,522 graphs from 328 slides were assigned to the training dataset, 3,985 graphs from 46 slides were assigned to the validation dataset, and 8,464 graphs from 95 slides were assigned to the testing dataset); the TCGA SCC dataset contains 33,998 graphs (23,667 graphs from 265 slides were assigned to the training dataset, 3,353 graphs from 37 slides were assigned to the validation dataset, and 6,978 graphs from 77 slides were assigned to the testing dataset). Graphs from the same slide were assigned to the same dataset to avoid data leakage. The independent NLST testing dataset contains 24,204 graphs from 327 ADC slides and 12,196 graphs from 169 SCC slides.

To train the ADC vs. SCC classification Ceograph, cross-entropy was used as loss function; Stochastic Gradient Descent (SGD) with learning rate = 0.0001 and momentum = 0.9 was used as optimizer. The model with highest classification accuracy in the validation dataset was selected and applied to the testing datasets. Majority voting among all graphs from labeled ROI was used to determine the histology subtype of a slide. Both patch-level and slide-level accuracies and receiver operating characteristic (ROC) curves were calculated to evaluate the classification performance.

### Ceograph For Malignant Transformation Prediction

To access the utility of Ceograph in predicting risk of malignant transformation of OPMD patients, we adapted the pathology image analysis pipeline proposed in Figure 1A to oral epithelial tissues (Figure 4A). To increase the receptive field of Ceograph, we increased the number of CSIGC layers to 4; to focus on epithelial cells, only features for epithelial stratum nuclei nodes were averaged through the Subgroup Mean Pooling layer (Supplemental Figure 8).

The prognostic Ceograph model was trained on the OPMD1 dataset and independently tested on the OPMD2 dataset. In the OPMD1 dataset, patients with short (CFS ≤ 42.3 months, n = 6) or long (CFS > 68.5 months, n = 17) survival outcome were assigned to a high- or low-risk group. Due to the limited number of available training samples, in each training epoch, one 2,048×2,048 pixel image patch containing at least 50 epithelial nuclei was randomly extracted from each patient; thus, image patches extracted from the same patient would differ across different training epochs. To construct the testing set, 100 2,048×2,048 pixels image patches were randomly picked up from each patient in the OPMD2 dataset, with each image patch containing at least 50 epithelial nuclei. The image patches in OPMD1 and OPMD2 were transformed into graphs individually.

To train the malignant transformation prognostic model, cross-entropy was used as loss function; SGD with learning rate = 0.0005 and momentum = 0.9 was used as optimizer. The model with highest classification accuracy in the OPMD1 set was selected. The probability of the graph predicted as high-risk group was used as the risk score of malignant transformation. The graph-level risk scores were averaged across all 100 patches for each individual patient to be summarized into patient-level risk scores. Patients were dichotomized into high- and low-risk groups with the patient-level risk score cutoff = 0.5.

### Ceograph for EGFR TKI Ttx Response Prediction

To assess the utility of Ceograph in predicting treatment response to EGFR TKI Ttx in patients with lung ADC, a deep learning-based pathology image analysis pipeline similar to Figure 1A was used (Figure 5A). To adapt for patient-level prognosis, the pipeline was modified in the following ways: 1) the categorical outputs were defined as non-benefitting and benefitting groups; 2) all connected graphs from one pathology slide were included in a single disconnected graph; 3) the subgroup mean pooling layer was replaced with a global mean pooling layer to take all cell types within the tumor microenvironment into consideration (Supplemental Figure 11).

The predictive Ceograph model was trained on the LCMC1 dataset and independently tested on the LCMC2 dataset. OS, defined as time from diagnosis of metastatic disease to death or last follow-up, was used as the outcome. In the LCMC1 dataset, patients with short (OS ≤ 31 month, #slides = 55) or long (OS > 31 months, #slides = 49) survival outcome were assigned to a non-benefitting or benefitting group, respectively (Supplemental Table 1). The OS cutoff was selected to ensure non-benefitting and benefitting groups are balanced.

To train the response prediction Ceograph model, cross-entropy was used as the loss function; AdaDelta(40) with scaling factor = 2 was used as the optimizer. The model with the highest classification accuracy in the LCMC1 dataset was selected. The probability of belonging to the benefitting group was used as the benefitting score. In the testing dataset, the benefitting scores were averaged into patient-level; then, the patients were dichotomized into benefitting and non-benefitting groups according to the median benefitting score.

### Ceograph Interpretation

The working mechanism can be interpreted in two aspects. First, since graph level supervision works as cellular level weakly-supervision, cellular level predictions can be computed to understand local contribution to the global prediction:

(1) $$x_{graph,k}=\frac{1}{N}\sum_{i=1}^{N}\;\;x_{nucleus,i,k}\;\forall k\in 1,\;2,\ldots ,K,$$

(2) $$P_{graph}=Softmax\left(x_{graph}\right),$$

(3) $$P_{nucleus,i}=Softmax\left(x_{nucleus,i}\right),$$

where K is the number of prediction categories, N is the number of nuclei involved in the Subgroup Mean Pooling Layer, and P represents the predicted probability vector. Mapping and visualizing $P_{nucleus}$ spatially enabled understanding of how local cell spatial organization patterns affect the whole graph determination (Figure 2D–E).

Second, feature-wise contribution to the graph prediction can be computed in a model agnostic way(33):

(4) $${Contribution}_{f}=\frac{\partial L}{\partial f},$$

(5) $$L=Cross\;Entropy\left(P_{graph},\;Presumptive\;category\;k\right),$$

where f represents either node (nucleus) or edge (cell-cell spatial interaction) feature of an individual node or edge, and L is an objective (loss) function. Hereby, partial derivative is used to conveniently calculate how increasing f by a small amount affects the objective function. A positive partial derivative indicates that increasing f results in a larger loss value; thus, feature f contributes negatively to the category k in this case. In practice, the partial derivative for all features could be calculated simultaneously, making this strategy a quick and efficient way to assess the contribution of each derivable feature. Mapping the node or edge level contributions back to the spatial locations enabled understanding of how local cell organizations contribute to the graph level prediction. A boxplot grouped by different nucleus or cell-cell connection types and across an entire dataset allowed for further comparison with pathological knowledge and identification of the key nucleus or cell-cell connection types and features that could be important biomarkers.

### Survival Analysis

Kaplan-Meier (KM) curves, log-rank tests, and Cox Proportional Hazard (CoxPH) models were used to evaluate the survival difference between two patient groups: between high- and low-risk groups to evaluate the performance of prognostic Ceograph in predicting risk of malignant transformation, and between EGFR TKI Ttx treated and non-treated patients in the benefitting group and non-benefitting group, respectively, to evaluate performance of predictive Ceograph in predicting response of EGFR TKI Ttx. ROC curves were plotted and AUC scores were calculated to evaluate the performance of the prognostic Ceograph in predicting 24- and 50-months cancer-free-survival probability for patients with OPMD, respectively. Multivariate CoxPH model was used to adjust for potential confounders, including age, gender, smoking status, surgery, and stage at initial diagnosis. The differences were considered significant when the two-tailed p-value was less than 0.05. Python libraries “scikit-learn” (v0.20.3), “torch” (v1.0.1.post2)(41), “torch-geometric” (v1.0.3) were used for graph creation and Ceograph implementation; the R library “survival” (v2.41–3) was used for survival analysis.

## Supplementary Material

Supplement 1

## Figures and Tables

**Table 1:** Algorithm of cell spatial interaction-conditioned graph convolutional layer (CSIGC).

	Algorithm details	Description
1	Input: layer ID i, node set with N nodes, node features $\text{X}\left(\text{i}\right)\in {\mathbb{R}}^{\text{N}\times x\left(\text{i}\right)}$, edge features $E\in {\mathbb{R}}^{\text{e}\times 3}$ (three edge features are edge type $t_{n,\;m}$, nuclear closeness $w_{n,\;m}$, and nuclear parallelism $a_{n,\;m}$), channel number of output layer c. Output: node features $\text{X}\left(\text{i}+1\right)\in {\mathbb{R}}^{\text{N}\times c}$	N: number of cells; X: nuclear morphological features; E: cell-cell interaction features.
2	**for** node n **in** N **do**	To describe a single cell niche
3	**# Step 1:** Calculate message from neighbors	Measure neighboring cells
4	**for** m **in** neighbors of node n **do**	With regard to each pair of the cell and one of its neighbors
5	Embedding $t_{n,\;m}$ to edge modulator $mo_{\text{n},\;\text{m}}\in {\mathbb{R}}^{\left(\text{x}\times \text{c}\right)}$ through edge type embedding $\mathrm{EM}\in {\mathbb{R}}^{36\times (x\times c)}$	Transfer categorical cell-cell interaction type into continuous features
6	Update ${mo}_{\text{n},\;\text{m}}$ m with ${\text{W}}_{\text{n},\;\text{m}}$, ${\text{a}}_{\text{n},\;\text{m}}$, through feature-wise linear modulation	Calculate cell-cell interaction-wise attentions
7	Reshape ${mo}_{\text{n},\text{m}}\in {\mathbb{R}}^{\left(\text{x}\times \text{c}\right)}$ as ${mo}_{\text{n},\text{m}}\in {\mathbb{R}}^{\text{x}\times \text{c}}$	
8	Calculate message $me_{\text{n},\;\text{m}}=X_{\text{n}}\times mo_{\text{n},\;\text{m}};me_{\text{n},\;\text{m}}\in {\mathbb{R}}^{1\times \text{c}}$	Apply attention to cell features
9	**# Step 2:** Calculate message from the self node	Measure the cell itself
10	$me_{\text{n}}=X_{\text{n}}\times \theta^{\text{i}};\;me_{\text{n}}\in {\mathbb{R}}^{1\times \text{c}}$	
11	**# Step 3:** Aggregate the messages from input layer i	Combine neighbors and self measurement together
12	Output features $X{\left(i+1\right)}_{\text{n}}=me_{\text{n}}+\text{Ave}\left(me_{\text{n},\;\text{m}}\right)$	
13	**# Step 4:** Activation and training-specific dropout layer	Add non-linearity for better network flexibility
14	$X{\left(i+1\right)}_{n}=\text{Dropout}(\text{ReLU}\left(X{\left(i+1\right)}_{n}\right)$, training flag)	
15	**end for**	

ReLU, Rectified Linear Unit.

**Table 2:** Performance comparison of different lung ADC vs. SCC classification models

Model	Training Set	Testing set	Slide-level AUC*
Bagging of multiple traditional machine learning models(15)	TCGA training set	TCGA testing set	0.75
Inception V1 at 40X(17)	TMA and TCGA training set	TMA and TCGA testing set	0.83
ResNet(18)	TCGA training set	TCGA testing set	0.857
Inception V3 at 20X(19)	TCGA training set	TCGA testing set	0.87
Inception V3 at 20X(16)	TCGA training set	NYU-LMC	0.905
**Logistic regression using HD-Staining derived image features** ^†^	TCGA training set	NLST	0.914
Inception V3 at 5X(16)	TCGA training set	NYU-LMC	0.933
PathCNN at 20X(20)	TCGA training set	TCGA testing set	0.957
**ResNet at 40X** ^†^	TCGA training set	NLST	0.966
**Ceograph** ^†^	TCGA training set	NLST	**0.999**

* Reported AUCs in independent testing dataset are recorded (if no independent testing result is reported, testing result in the same dataset is used in the table instead). If multiple AUCs are reported, average value among all datasets is used. For the purpose of comparable comparison, in reporting AUC of the Inception V3 model(16), only frozen sections and formalin-fixed paraffin-embedded (FFPE) sections are included.

† Same data settings are used as direct comparison for Ceograph.

ADC, adenocarcinoma; AUC, Area under Curve; SCC, squamous carcinoma; NLST, National Lung Screening Trial; NYU-LMC, New York University Langone Medical Center dataset; TCGA, The Cancer Genome Atlas; TMA, Tissue Microarrays.

## Data Availability

Pathology images that support the findings of this study were available online in NLST (https://biometry.nci.nih.gov/cdas/nlst/) and The Cancer Genome Atlas Lung Adenocarcinoma (TCGA-LUAD, https://wiki.cancerimagingarchive.net/display/Public/TCGA-LUAD).
