# Supplementary material for: Deep Learning of Cell Spatial Organizations Identifies Clinically Relevant Insights in Tissue Images
Source: Res Sq. 2023 Jul 4:rs.3.rs-2928838. Preprint. [Version 1] doi: 10.21203/rs.3.rs-2928838/v1 (PMC10350240; doi:10.21203/rs.3.rs-2928838/v1)
Supplement: Supplement 1 [file NIHPPrs2928838v1-supplement-1.pdf]

Supplemental Tables and Figures

Supplemental Table 1 Patient characteristics of LCMC1 training set and LCMC2 testing set.

	Training (LCMC1)		Validation (LCMC2)	P value
	Benefitting	Non-benefitting		
# EGFR mutated	50	48	126	
# EGFR Ttx treated	50	48	90	
# Biopsy slides	64	51	137	
Age (year)	62.4 ± 9.6	59.5 ± 10.4	62.7 ± 10.1	0.16
Gender (%)				0.028
Male	8 (16%)	11 (23%)	44 (35%)	
Female	42 (84%)	37 (77%)	82 (65%)	
Smoking status (%)				0.086
Current	0 (0%)	3 (6%)	3 (2%)	
Former	23 (46%)	13 (27%)	57 (46%)	
Never	27 (54%)	32 (67%)	65 (52%)	
Surgery received? (%)				0.0038
No	18 (36%)	35 (73%)	83 (66%)	
Yes	32 (64%)	13 (27%)	41 (33%)	
Unknown	0 (0%)	0 (0%)	2 (2%)	
Stage at diagnosis (%)				0.068
I	6 (12%)	2 (4%)	5 (4%)	
II	3 (6%)	2 (4%)	3 (2%)	
III	11 (22%)	7 (15%)	10 (8%)	
IV	29 (58%)	36 (75%)	105 (83%)	
Unknown	1 (2%)	1 (2%)	3 (3%)	

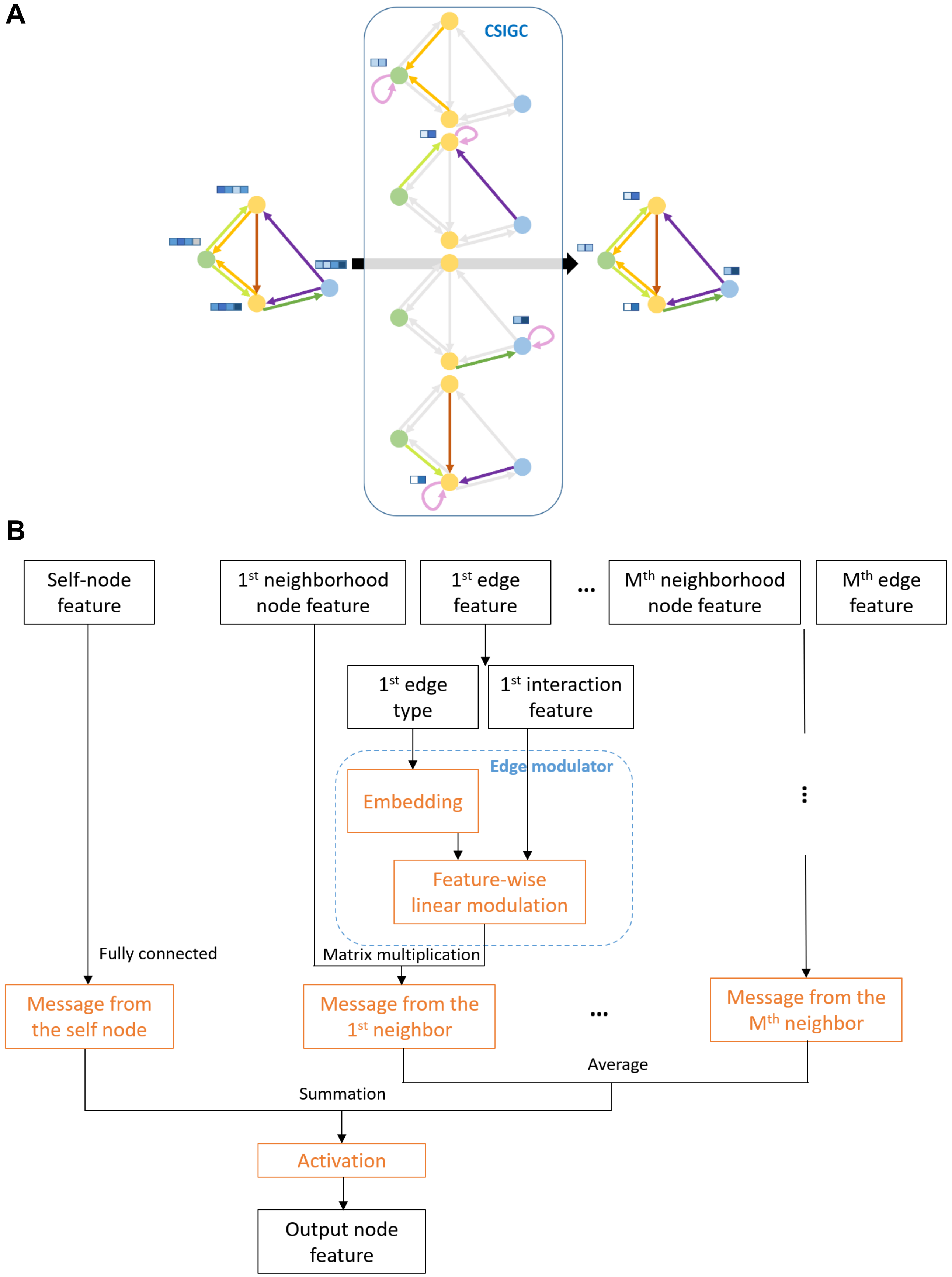

Supplemental Figure 1: Illustration of the cell spatial interaction-conditioned graph convolution (CSIGC) algorithm. (A) The feed-forward process of one CSIGC Layer, a building

block of Ceograph. The input is an example graph consisting of 4 nodes, each with a length-4 feature vector. During the CSIGC, each node receives and integrates messages from itself and its neighbors, which are calculated using the input node features and edge features. The integrated message becomes node features and serves as inputs for the next layer. Different colors indicate different nuclei or edge types. **(B)** Flowchart of the detailed computing process of one node feature (referred to as the self-node) through one CSIGC Layer. Orange boxes indicate the learnable layers involved in back-propagation.

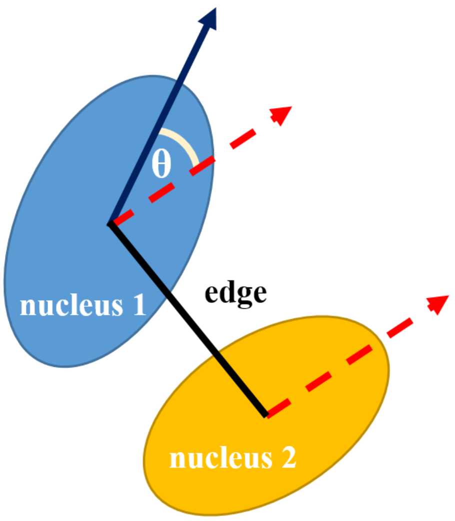

Supplemental Figure 2 Illustration of the definition of "parallelism". Absolute value of Cosine θ is used to evaluate the orientation parallelism between a pair of nuclei.

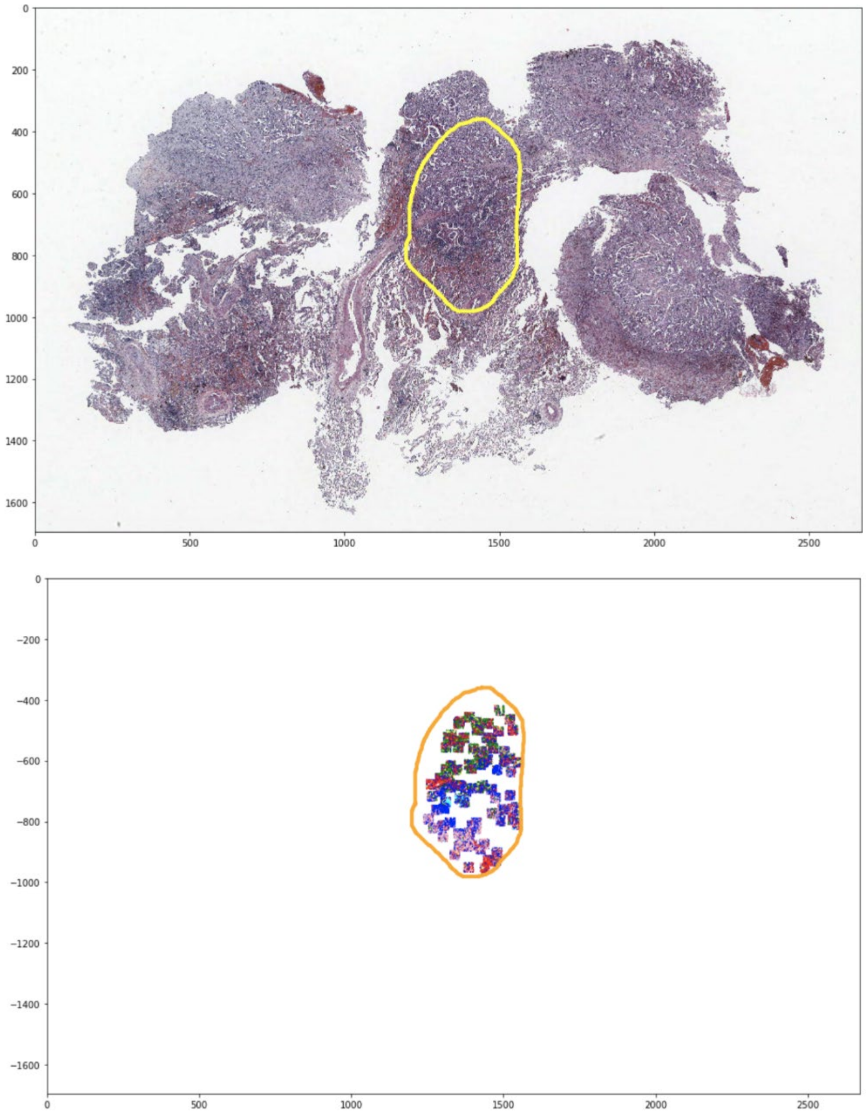

Supplemental Figure 3 Illustration of patch extraction from Region of Interest (ROI) labeled by pathologist. Upper panel: pathology slide with ROI annotated in yellow; **Lower panel:** 100 patches randomly extracted from the ROI, stained by HD-Staining.

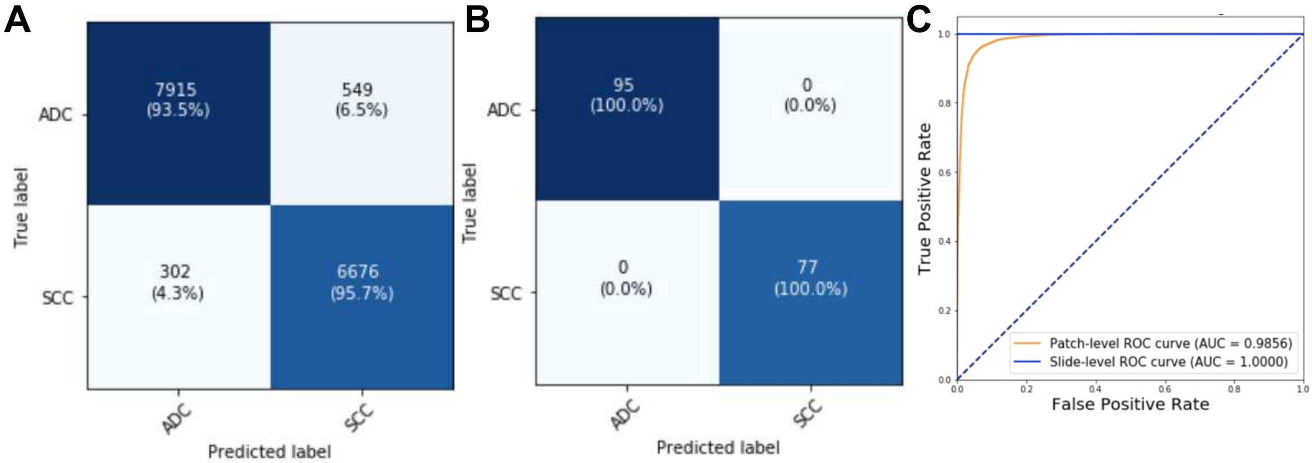

Supplemental Figure 4 Ceograph classification performance in the TCGA testing dataset. image patch-level confusion matrix **(A)**, slide-level confusion matrix **(B)**, ROC curve **(C)**.

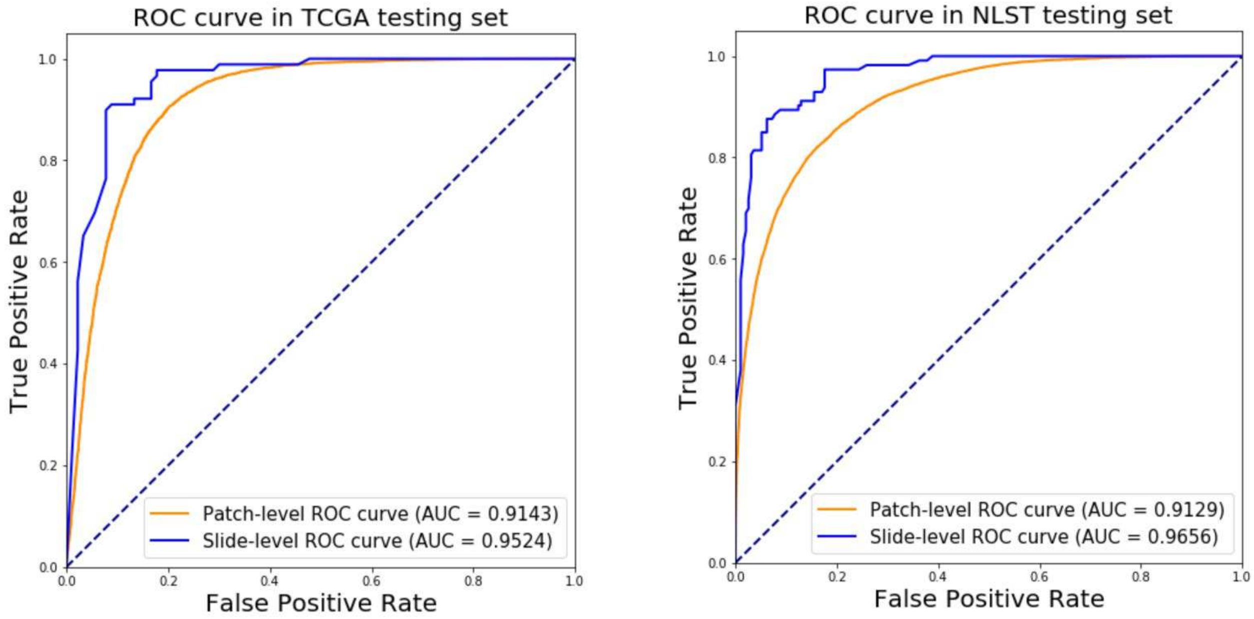

Supplemental Figure 5 ROC curve of using ResNet101 model to classify lung adenocarcinoma vs. squamous carcinoma. The model is trained and tested using exactly the same image patches as Ceograph model.

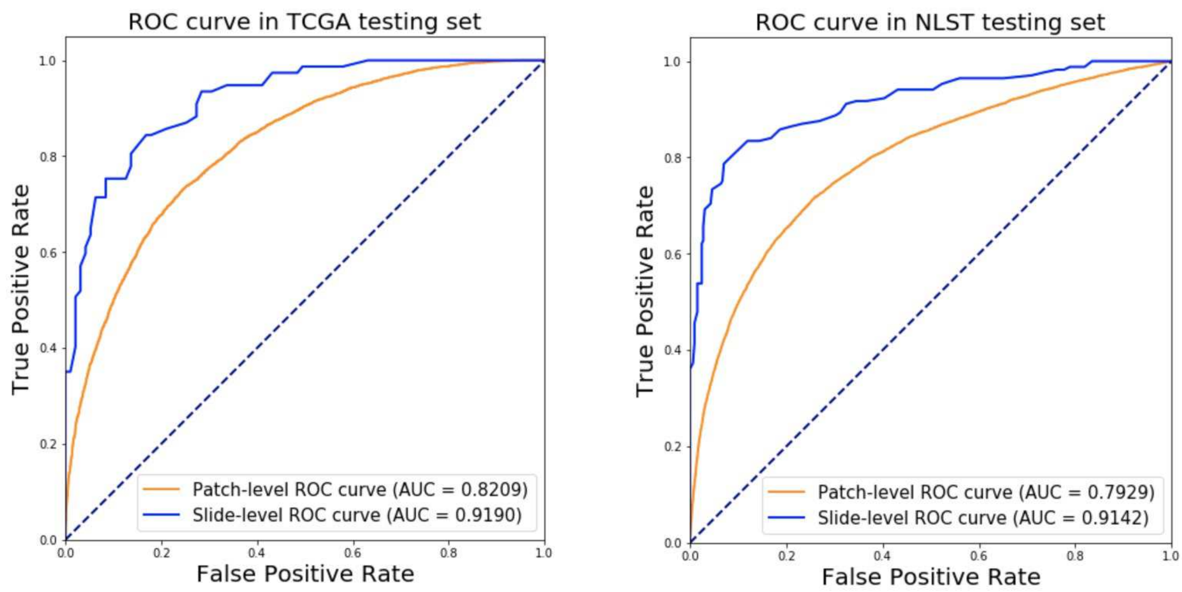

Supplemental Figure 6 ROC curve of using logistic regression model to classify lung adenocarcinoma vs. squamous carcinoma. The model is trained and tested using exactly the same datasets as the Ceograph model. The input features are node features averaged on tumor nuclei.

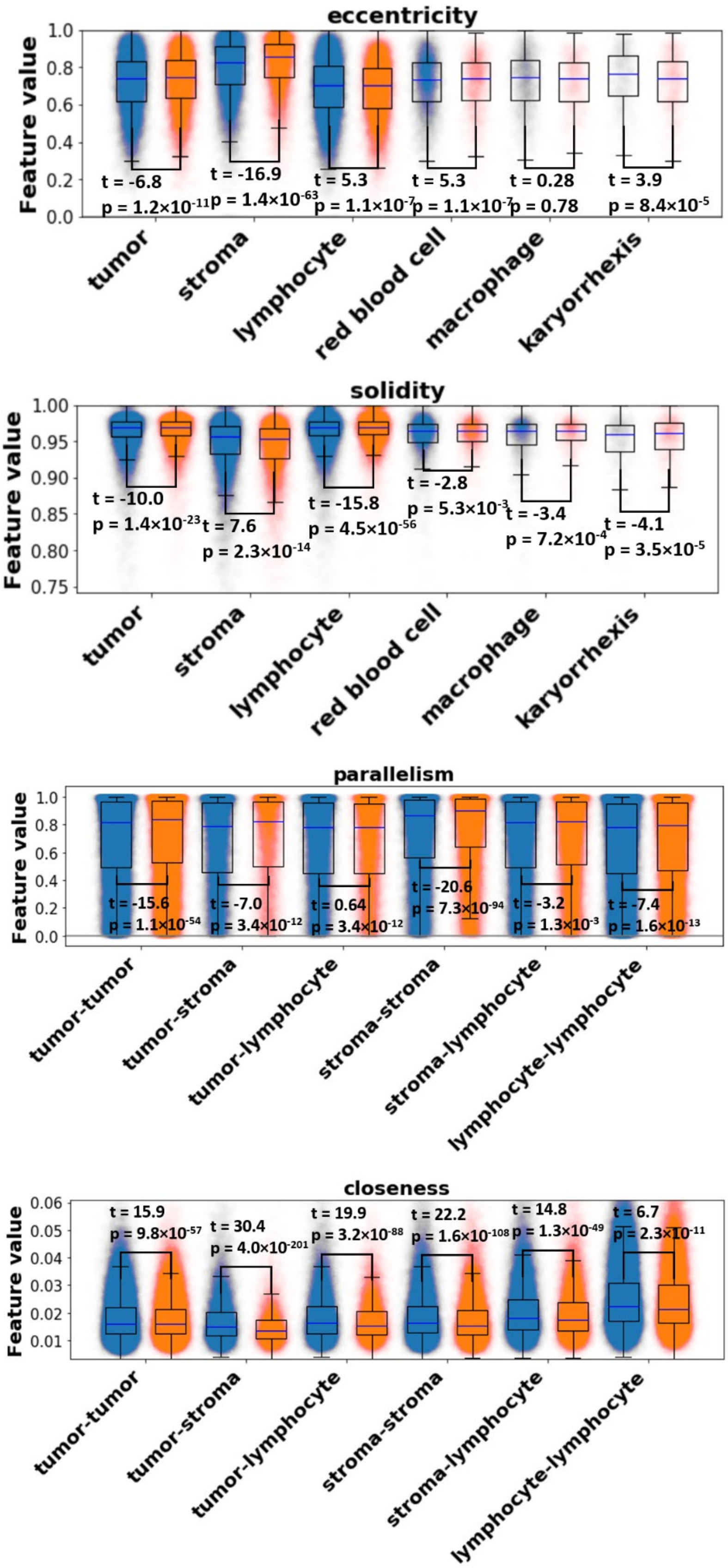

Supplemental Figure 7 Comparison of image features between ADC and SCC in the NLST dataset. Blue, ADC; orange, SCC

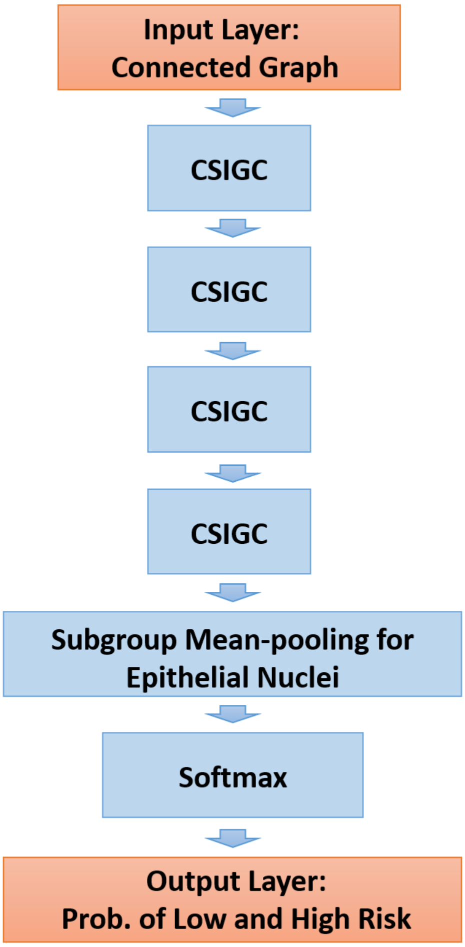

Supplemental Figure 8 Prognostic Ceograph structure. CSIGC, cell spatial interaction-conditioned graph convolutional layer; prob., probability.

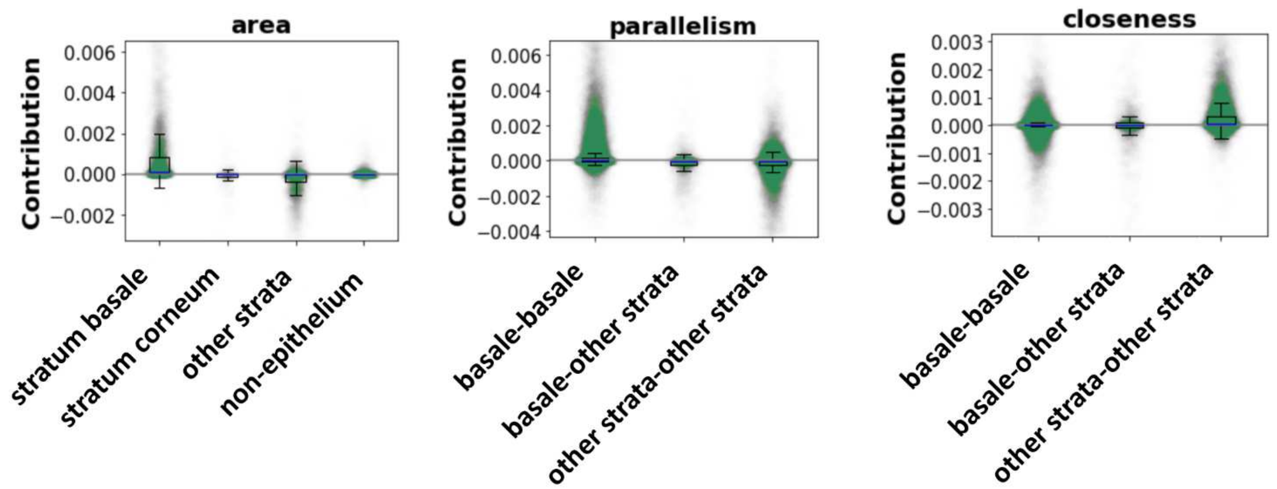

Supplemental Figure 9 Boxplots to summarize feature contributions across the entire OPMD1 dataset. Positive value indicates contribution to high-risk group, while negative value indicates contribution to low-risk group.

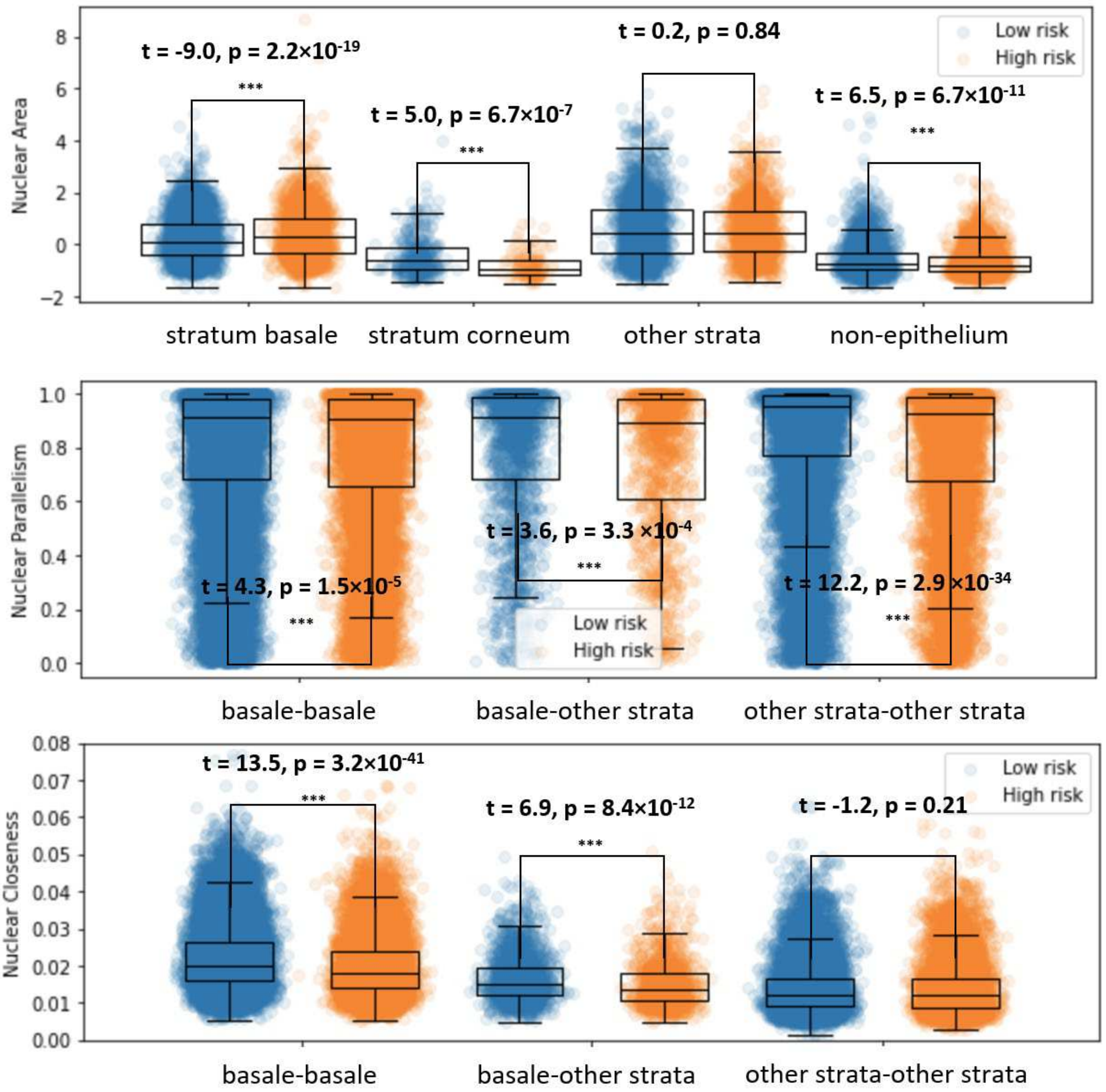

Supplemental Figure 10. Comparison of nuclear and edge features between low- and high-risk patients in the OPMD1 dataset, stratified by nuclear and edge types.

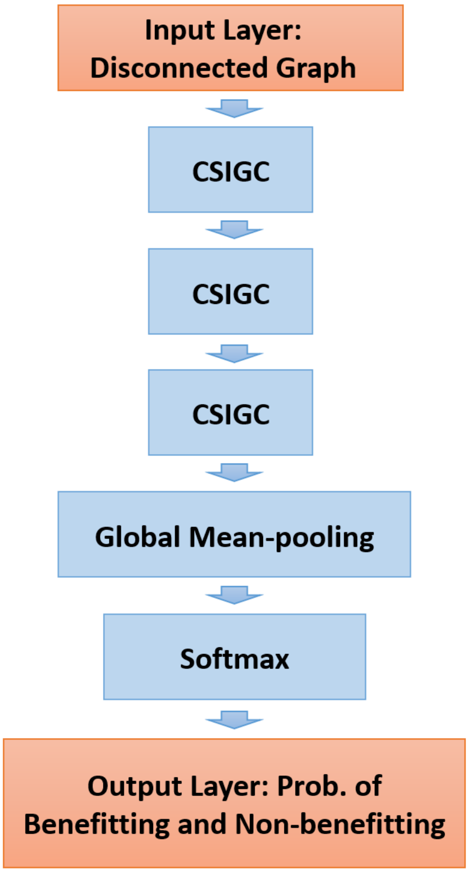

Supplemental Figure 11 Predictive GCN structure. CSIGC, cell spatial interaction-conditioned graph convolutional layer; prob., probability.

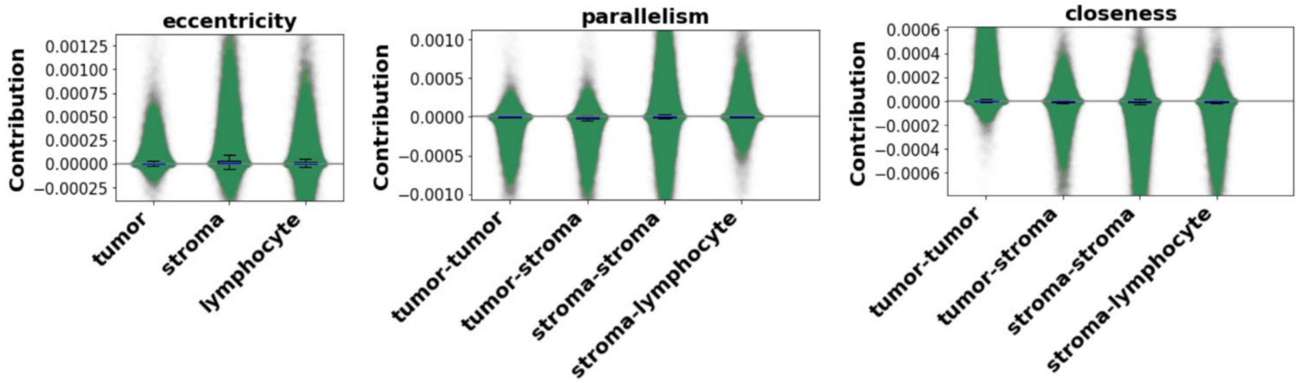

Supplemental Figure 12 Boxplots to summarize feature contributions across the entire LCMC1 dataset. Positive value indicates contribution to non-benefitting group, while negative value indicates contribution to benefitting group.

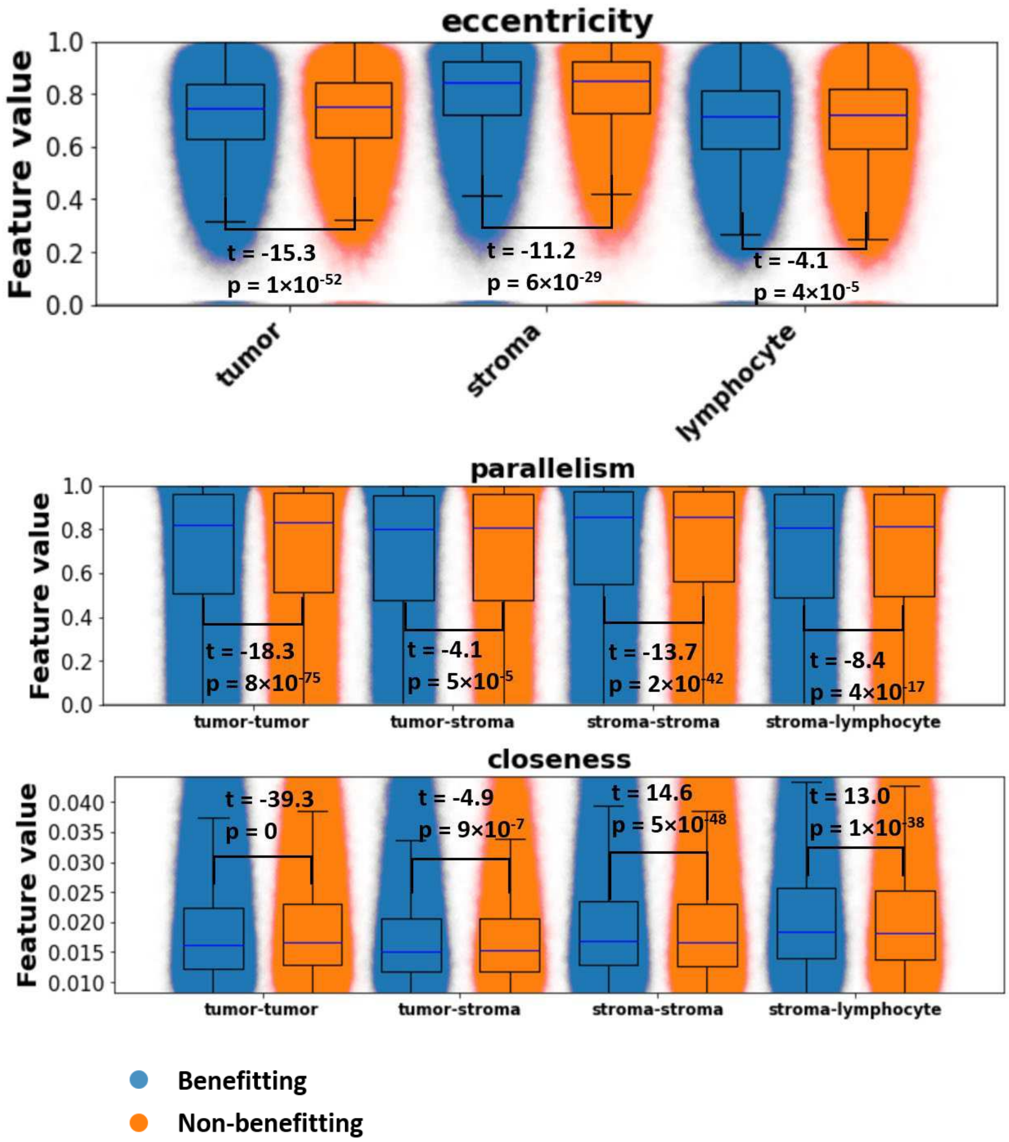

Supplemental Figure 13 Comparison of nuclear and edge features between benefitting and non-benefitting patients in the LCMC1 dataset who carry *EGFR* mutation and received EGFR Targeted therapy, stratified by nuclear and edge types.
